# Human DREF/ZBED1 is a nuclear protein widely expressed in multiple cell types derived from all three primary germ layers

**DOI:** 10.1371/journal.pone.0205461

**Published:** 2018-10-10

**Authors:** Simone Valentin Hansen, Sofie Traynor, Henrik Jørn Ditzel, Morten Frier Gjerstorff

**Affiliations:** 1 Department of Cancer and Inflammation Research, Institute for Molecular Medicine, University of Southern Denmark, Odense, Denmark; 2 Department of Oncology, Odense University Hospital, Odense, Denmark; 3 Academy of Geriatric Cancer Research (AgeCare), Odense University Hospital, Odense, Denmark; National Eye Centre, UNITED STATES

## Abstract

*Drosophila* DNA replication-related element binding factor (DREF) is a transcription regulatory factor that binds the promoters of many genes involved in replication and cell proliferation and is required for normal cell cycle progression. Human DREF/zinc finger BED domain-containing protein 1 (ZBED1), an orthologue of *Drosophila* DREF, also has DNA binding activity, but its cellular functions remain largely uncharacterized. Herein, we show that ZBED1 is a chromatin-associated nuclear protein with a wide expression profile in human tissues from all three primary germ layers. For instance, ZBED1 was expressed in mesodermal-derived epithelial cells of the reproductive system and urinary tract, in endodermal-derived epithelial cells throughout the gastrointestinal tract, and in epidermal epithelium from the ectoderm. ZBED1 was also expressed in connective tissue and smooth muscle cells of multiple organs. To investigate whether ZBED1 is implicated in cell proliferation, similar to *Drosophila* DREF, we compared the tissue distribution of ZBED1 to that of the proliferation marker Ki-67. ZBED1 and Ki-67 were co-expressed in many epithelial tissues, but ZBED1 expression extended widely beyond that of Ki-67-positive cells. In other tissues, ZBED1 expression was more restricted than Ki-67 expression. These results suggest that ZBED1 is not a cell proliferation-associated factor such as *Drosophila* DREF, and our study adds to the cumulative understanding of the functions of ZBED1 in human cells and tissues.

## Introduction

The zinc finger BED domain-containing protein 1 (ZBED1) is also known as DNA replication-related element binding factor (DREF) and was first discovered in *Drosophila*. *Drosophila* DREF (dDREF) contains a BED zinc finger domain at the N-terminal of the protein, which has DNA binding activity and is responsible for binding to the consensus sequence 5’-TATCGATA, also called a DNA replication-related element (DRE) [1, 2]. When DREs are located several hundred base pairs upstream of the transcriptional start site, dDREF can function as a traditional transcription factor [3, 4], and it can also function as part of the basal transcriptional machinery together with TBP Related Factor 2 (TRF2) [5]. dDREF is involved in chromatin organization and epigenetic regulation through activation of the transcription of key subunits of the BRM chromatin-remodelling complex [6], by direct association with the XNP/dATRX chromatin remodelling complex [7], by interacting with the Mi-2 chromatin remodelling protein [8] and by antagonizing the boundary element-associated factor (BEAF) [9]. dDREF also activates the transcription of the *Drosophila* histone methyltransferase, nuclear receptor-binding SET domain protein (NSD) [10] and may play a role in telomere maintenance [8].

Additionally, dDREF is instrumental for cell proliferation in enhancing the expression of several genes important for DNA replication and cell cycle progression, including replication factor C 1 (rcf1) [11], S-Phase Kinase Associated Protein 1-related A (skpA) [12], E2F [13], cyclin A [14], DNA polymerase α and proliferating cell nuclear antigen (PCNA) [2, 3, 15]. Gene knockdown demonstrated that dDREF is required for cells to efficiently progress through the late G1 and S phase of cell cycle, further demonstrating its importance in cell proliferation [3]. dDREF also regulates the transcription of several genes involved in replication and transcription of mitochrondrial DNA [8], and is involved in control of protein synthesis by positive regulation of genes required for ribosome biogenesis [8].

Studies suggest that dDREF play a role in tumorigenesis by regulating tumor suppressors and oncogenes. The p53 tumor suppressor is positively regulated by dDREF, and DRE and DRE-like binding sequences have been found in the promoter regions of other tumor suppressor genes such as Breast Cancer 2 (Brca2), Von Hippel-Lindau (VHL) and Retinoblastoma (Rbf) [8]. Additionally, dDREF regulates both tumor suppressors and oncogenes of the Hippo pathway [8]. Other processes indirectly related to tumorigenesis such as DNA damage repair [16] and the antioxidant defence [17] are also regulated by dDREF.

Expression of dDREF is regulated by the Myc proto-oncogene, which has various functions including inhibition of differentiation and induction of growth and proliferation [8]. Studies also suggest that Myc is required for expression of dDREF in various development-associated pathways in *Drosophila* [8]. dDREF has further been associated with development by regulating the JNK and epidermal growth factor receptor (EGFR) pathways, which are involved in thorax development and vein formation, respectively [8]. Moreover, it has been suggested that dDREF functions downstream of the Target-of-Rapamycin (TOR) pathway, which regulates cell and tissue growth in response to nutritional conditions [8].

The human homolog of DREF is called ZBED1, or human DNA replication-related element binding factor (hDREF), and it shares 22% amino acid identity with the *Drosophila* homolog [18]. Like dDREF, ZBED1 contains a N-terminal BED zinc finger domain with DNA binding activity [1, 19] and a ATC domain at the C-terminal, which has dimerization activity and is responsible for its self-association, which is necessary for DNA binding [20]. In vitro, ZBED1 binds to the human DRE consensus sequence 5’-TGTCG(C/T)GA(C/T)A [18], which is partly identical to the *Drosophila* DRE sequence. A few studies have further suggested that ZBED1 and dDREF have overlapping functions in regulation of cell proliferation. For instance, ZBED1 expression is induced in the G1-S phase of the cell cycle and up-regulates expression of the histone H1 gene, which is stringently coupled with DNA replication [18]. Furthermore, ZBED1 stimulates the transcription of some ribosomal protein genes [21], and is also involved in epigenetic regulation through mediating SUMOylation of Mi2α, an ATP-dependent DNA helicase that is part of the nucleosome remodeling and deacetylation (NuRD) complex [22]. This modification may cancel transcriptional repression by NuRD and thereby be important for transcriptional regulation of ZBED1 target genes [22].

To further understand the role of ZBED1 in human cells and tissues, we have characterized the distribution of ZBED1 protein in a large number of tissues and organs and correlated it with Ki-67 expression. Our results demonstrate wide expression of ZBED1 and suggest that ZBED1 expression is not associated with cell proliferation.

## Materials and methods

### Cell culture

A375 cells (obtained from ATCC, Wesel, Germany) were grown in DMEM medium (Sigma Aldrich, Soborg, DK) with 10% FBS (Sigma Aldrich) and penicillin/streptomycin. Cells were kept at low passage and cultured for no more than 3 months.

### Immunofluorescence analysis of ectopic expression of ZBED1 in A375 cells

A pLX305 plasmid for expression of ZBED1 (NP_004720.1) with C-terminal V5-tag from a CMV promoter was obtained from the DNA Resource Core at Harvard Medical School (Boston, MA, USA). The plasmid was introduced into A375 cells using Optifect according to the manufacturer’s recommendations (Life Technologies, Naerum, Denmark). Cells were stained with monoclonal rabbit anti-V5 antibody (clone D3H8Q; Cell Signaling, Leiden, The Netherlands) and DAPI DNA stain, as previously published [23].

### Western blotting

Cells were harvested and extracted in RIPA buffer with protease inhibitors (Complete Protease Inhibitor Cocktail, Sigma Aldrich). Lysates were subjected to gel electrophoresis and blotted onto membranes as previously described [24]. Membranes were incubated with anti-ZBED1 antibody (clone OTI5A2; Cat. No.: TA505044; Origene Rockville, MD, USA) diluted 1/1000 in PBS, 1% BSA and subsequently with poly goat anti-mouse immunoglobulin HRP (DAKO Cytomation). Blots were developed with Clarity Western ECL Substrate (BioRad, Copenhagen, Denmark).

### Human tissue samples

Human tissues samples were obtained from the tissue bank at the Department of Pathology, Odense University Hospital (Odense, Denmark) with the approval of the Regional Committees on Health Research Ethics for the Region of Southern Denmark (Ref. No. VF20050069). Samples were from surgically-excised, histologically-normal specimens from at least two individuals. Tissue samples were fixed in 4% formaldehyde for 48 hours, dehydrated and embedded in paraffin.

### Immunohistochemistry

Immunohistochemistry was performed at the Department of Pathology, Odense University Hospital (Odense, Denmark). Sections of tissues were cut, deparaffinized and treated with 1.5% H_2_O_2_ in Tris-buffered saline (pH 7.5) for 10 minutes to block endogenous peroxidase activity, rinsed in distilled H_2_O, demasked for antigen retrieval and washed in TNT buffer (0.1 m Tris, 0.15 m NaCl, 0.05% Tween-20, pH 7.5). Different antigen retrieval protocols were initially evaluated, including microwave boiling for 15 minutes in (1) T-EG buffer (10 mm Tris, 0.5 mm EGTA, pH 9.0), (2) 10 mm citrate buffer (pH 6.0), or (3) Dako Target retrieval solution (Dako S1699), or proteolytic treatment using (4) 0.05% protease type XIV (pronase E, Sigma, cat. no. P5147) in TBS (pH 7.0) for 15 minutes at 37°C or (5) 0.4% pepsin (Sigma, Cat. No.: P7012) in 0.01 m HCl for 20 minutes at 37°C. The microwave boiling in T-EG buffer for 15 minutes was found to be the optimal antigen retrieval method.

Sections were incubated with mouse monoclonal anti-ZBED1 (clones OTI5A2, Cat. No.: TA505044 and OTI1F7, Cat. No.: TA505043, Origene Rockville) or mouse monoclonal anti-Ki-67 (clone 30–9, Ventana, Tucson, AZ, USA) diluted in antibody diluent (S2022, DAKO Cytomation, Glostrup, Denmark) for one hour at RT. Subsequently, sections were washed with TNT and incubated with EnVision Flex/HRP+ for 30 minutes, followed by another wash with TNT. The final reaction product was visualized by incubating with 3,3′-diaminobenzidine (DAB)+ substrate-chromogen for 10 minutes, followed by washing with H_2_O and counterstaining of sections with Mayers hematoxylin before mounting in AquaTex (Merck Inc., Whitehouse Station, NJ, USA).

Imaging was performed with a PlanApo N 60X/1.42 oil objective fitted on an Olympus IX73 microscope using CellSens Entry software (Olympus).

## Results

### Validation of ZBED1 antibodies for immunohistochemical staining

**Fig 1:**
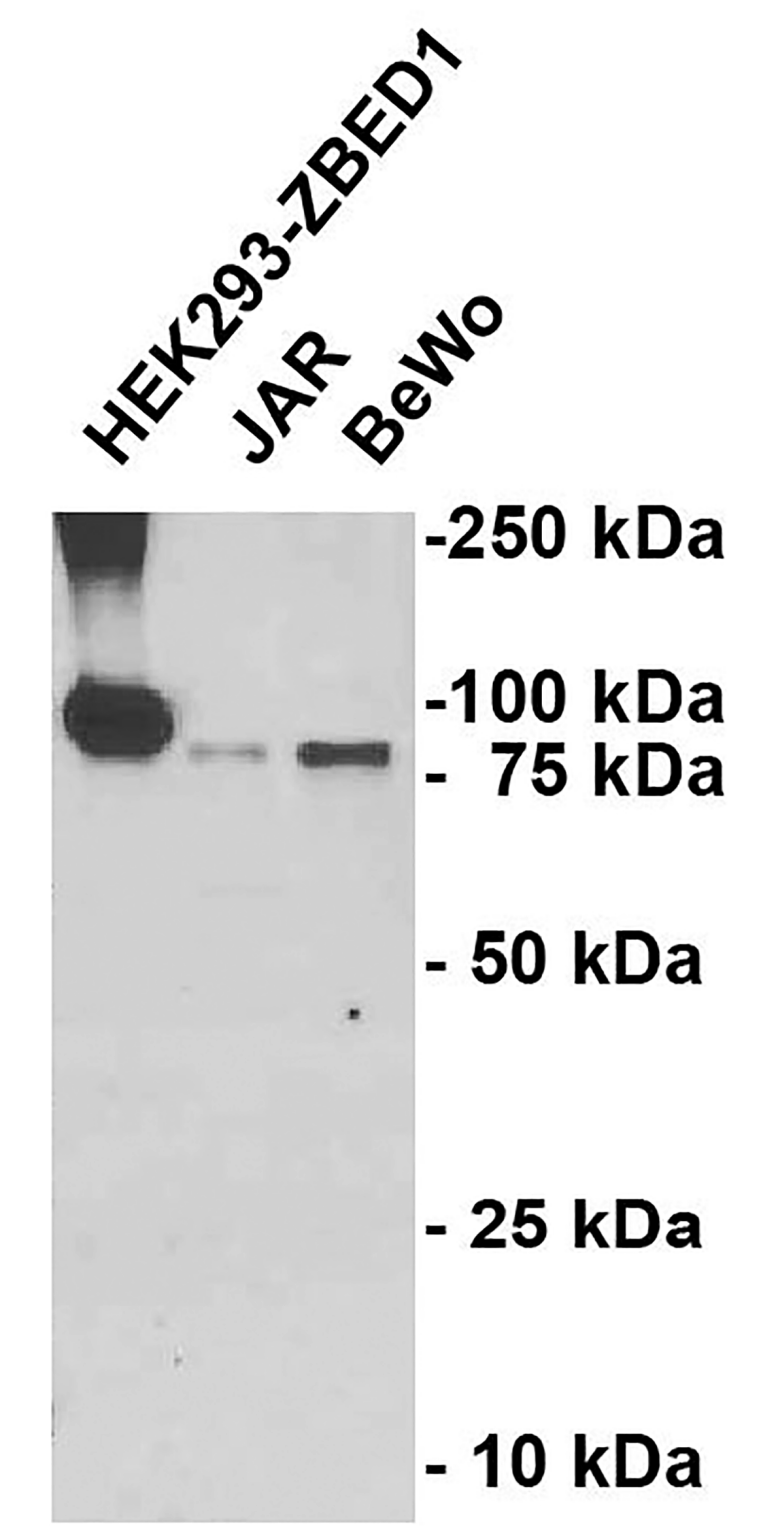
Validation of ZBED1 antibody specificity by Western blotting. The OTI5A2 antibody specifically recognized a band in JAR and BeWo cell lysates, corresponding to the predicted size of ZBED1, and slightly smaller than the band detected in HEK293 cells with ZBED1-C-MYC/DDK-tag overexpression.

To investigate ZBED1 expression in human tissues by immunohistochemistry, we obtained two monoclonal antibodies (OTI5A2 and OTI1F7) raised against full length recombinant ZBED1. The specificity of OTI5A2 was validated using Western blotting, where it specifically recognized a 78-kDa band corresponding to the theoretical size of ZBED1 in BeWo and JAR cell lysates (Fig 1). OTI1F7 did not did not work for Western blotting under the applied conditions.

**Fig 2:**
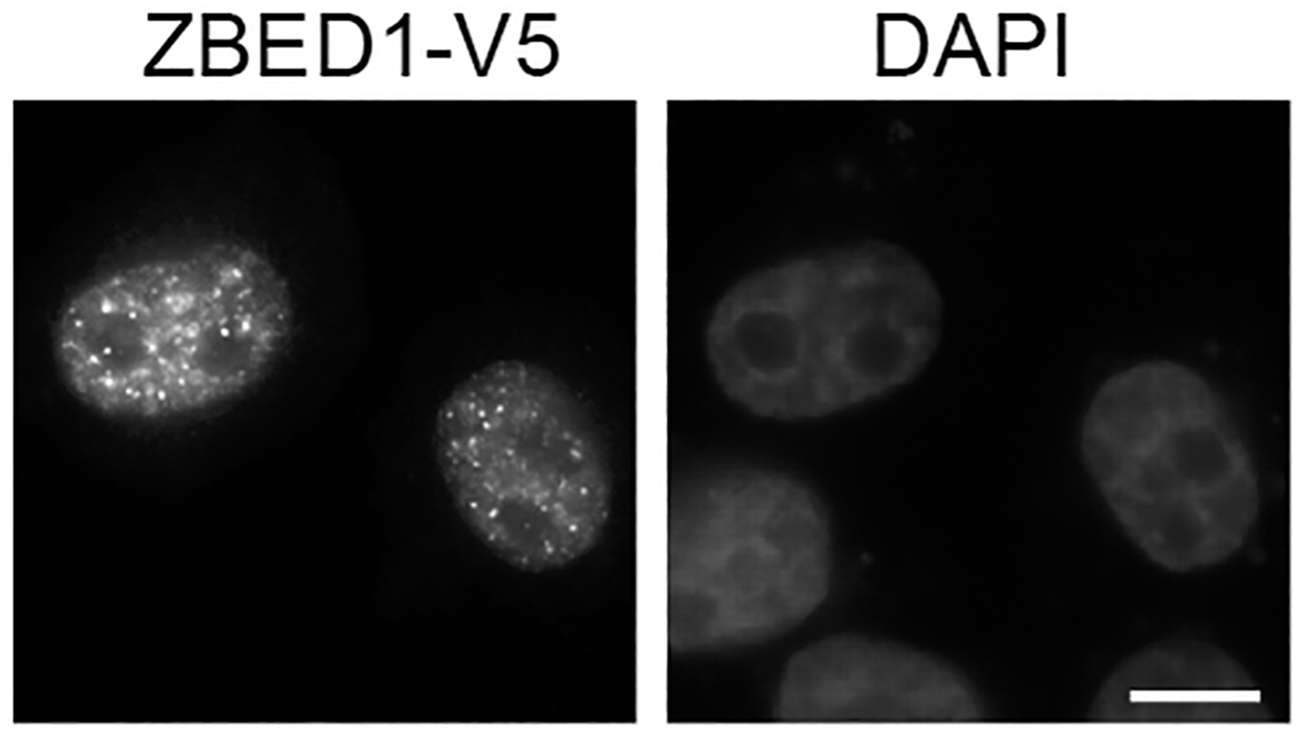
ZBED1 is a nuclear protein. ZBED1 was ectopically expressed in A375 cells with a C-terminal V5-tag for immunostaining. DAPI was used for staining of chromatin. Scale bar = 10 μM.

We then optimized conditions for immunohistochemical staining of tissue sections using these two antibodies. Five different antigen retrieval techniques were tested, including microwave heating with different buffers and treatment with protease. For both antibodies, microwave heating in T-EG buffer resulted in specific staining of cell nuclei in accordance with published results demonstrating localization of DREF in the nucleus of *Drosophila* cells [9] and cultured human cells (Fig 2). The two ZBED1 antibodies exhibited highly similar staining patterns in a panel of human normal tissues (representative immunostainings with OTI5A2 are shown in the figures of this study).

### ZBED1 expression in the organs of the digestive system

**Fig 3:**
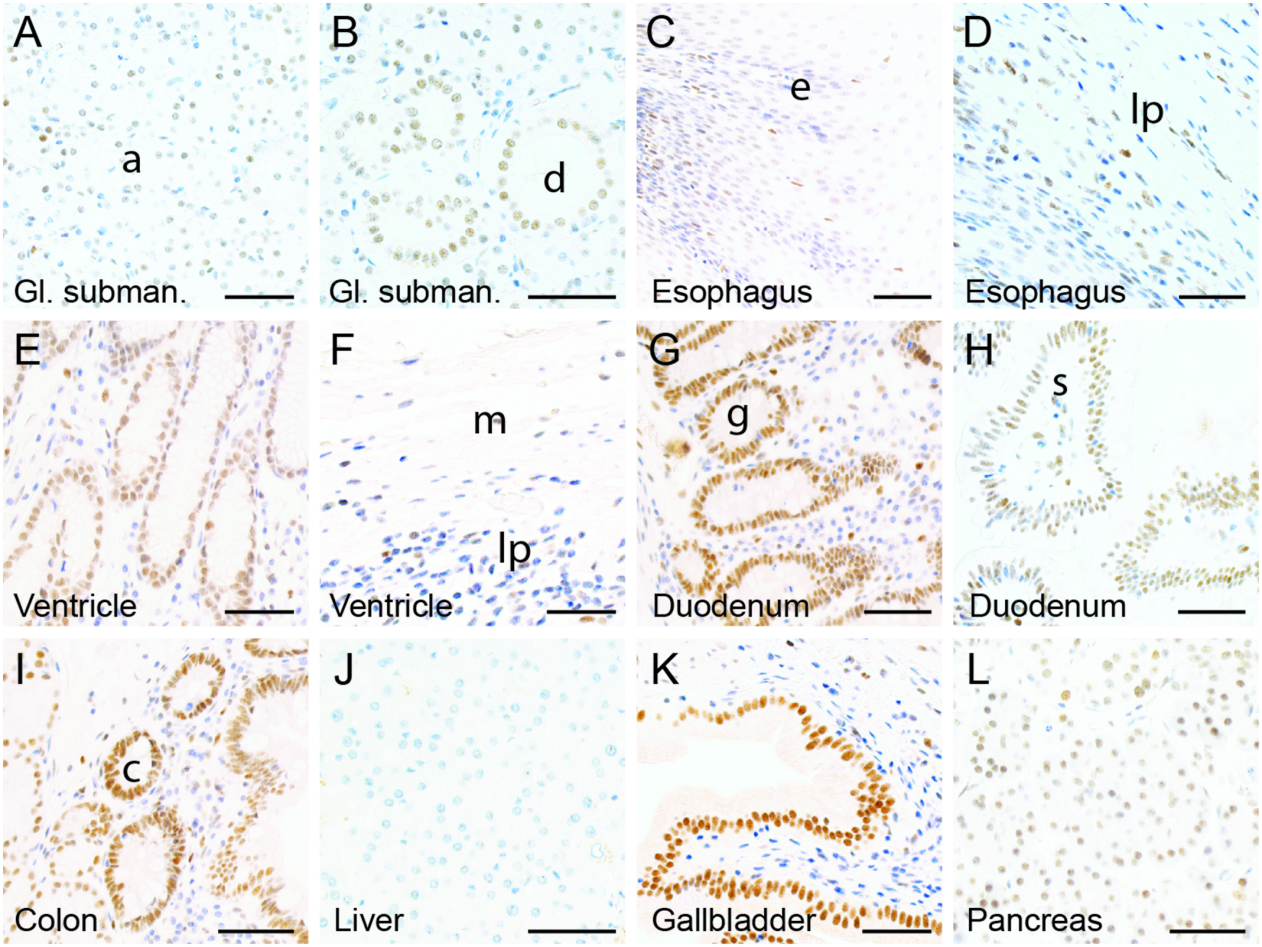
Expression of ZBED1 in the digestive system. (A-B) Glandular submandibularis, (C-D) esophagus, (E-F) ventricle, (G-H) duodenum, (I) colon, (J) liver, (K) gallbladder, and (L) pancreas. Abbreviations: a = acinar cells, d = ductal cells, e = epithelial cells, lp = lamina propria, m = muscularis; g = glandular cells, s = surface epithelial cells, c = crypts. Scale bars = 50 μM.

The expression of ZBED1 was investigated in representative tissues of the digestive system (Fig 3). In glandular submandibularis, ZBED1 was detected in all cells of the serous acini, intercalated ducts and striated ducts, while cells of mucous acini appeared negative (Fig 3A). In the esophageal tract, ZBED1 was expressed in cells of the basal layers of the stratified squamous non-keratinizing epithelium, but not in the outer layers (Fig 3B). ZBED1 expression was also detected in a subset of cells in the lamina propria and lamina muscularis, albeit at lower levels (Fig 3C). In stomach, ZBED1 was highly expressed in epithelial cells of the gastric gland (Fig 3D) and, to a lesser extend, in the surface-lining cells and some cells of the lamina propria (not shown). In the duodenum, both surface absorptive cells and goblet cells were positive (Fig 3E), as were ductal cells and cells in the glands of Brunner (Fig 3F). Similarly, both glandular and goblet cells of the colon were positive (Fig 3G-H). In duodenum and colon, ZBED1 expression was also demonstrated in subsets of cells of the lamina propria and smooth muscle layers (Fig 3I). In the liver, hepatocytes and Kupffer cells were completely negative for ZBED1 (Fig 3J), while epithelial cells of the gall bladder and a subset of the subjacent connective tissue cells were highly positive (Fig 3K). In the pancreas, we found that cells of the islets of Langerhans were weakly positive, as were acinar cells (Fig 3L).

### ZBED1 expression in immune organs

**Fig 4:**
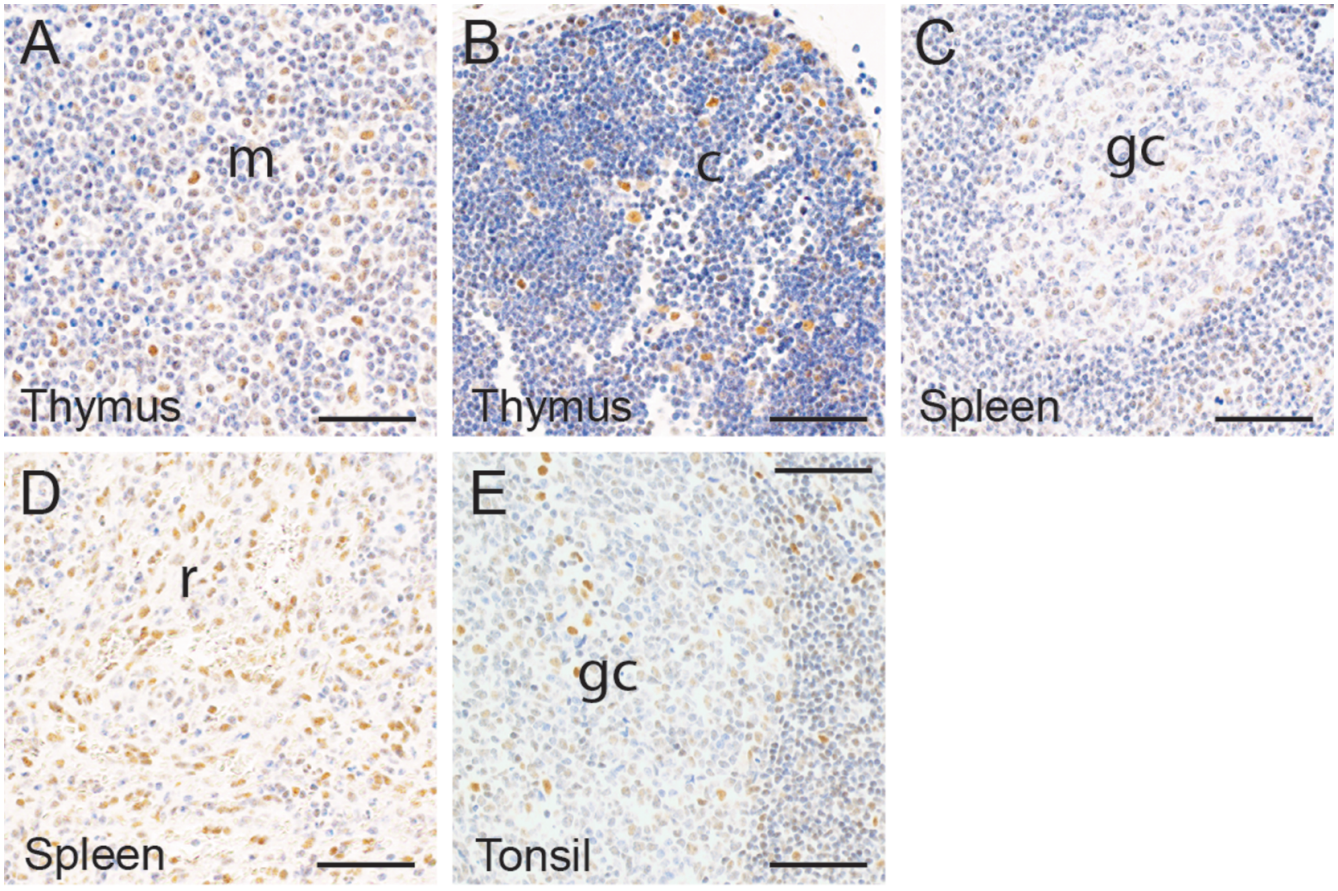
ZBED1 expression in immune organs. (A-B) thymus, (C-D) spleen, and (E) tonsil. Abbreviations: m = medulla, c = cortex, gc = germinal center, r = red pulp. Scale bars = 50 μM.

In the thymus, ZBED1 was weakly expressed in a large proportion of cells of the cortex (Fig 4A), and highly expressed in a small number of medullary cells (Fig 4B). The number of positive cells and their localization suggested that they were epithelial reticular cells and that thymocytes were negative. In the spleen, low expression was found in a subset of white pulp cells (Fig 4C), and medium expression in a subset of cells in the red pulp (Fig 4D). The lymphatic nodules of tonsils contained a small number of weakly positive cells (Fig 4E), whereas a high level of expression was found in numerous cells of the stroma surrounding the crypts (Fig 4F). Similar to the thymus, the staining pattern of ZBED1 in the spleen and tonsil suggested that only non-lymphocyte cell types were positive.

### ZBED1 expression in reproductive organs and placenta

**Fig 5:**
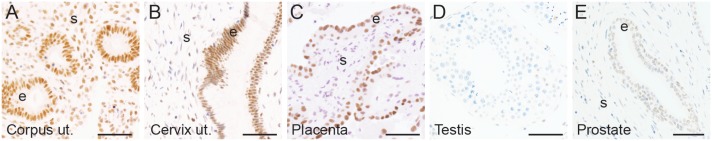
Expression of ZBED1 in reproductive tissues. (A) corpus uterus, (B) cervix uterus, (C) placenta, (D) testis, and (E) prostate. E = epithelial cells, s = stroma cells. Scale bars = 50 μM.

In the female reproductive system, strong expression was detected in all epithelial cells and in a subset of stromal cells of the cervix uterus (Fig 5A) and corpus uterus (Fig 5B). In placenta, ZBED1 was highly expressed in the trophoblast epithelial layer of the blastocyst. Expression was seen in both the inner cytotrophoblast and in the outer syncytiotrophoblast, but was most pronounced in the latter (Fig 5C).

In contrast, ZBED1 was not detected in the male reproductive system, including cells of the testes tubules and connective tissue (Fig 5D), and stromal or epithelial cells of the prostate (Fig 5E).

### ZBED1 expression in other tissues

**Fig 6:**
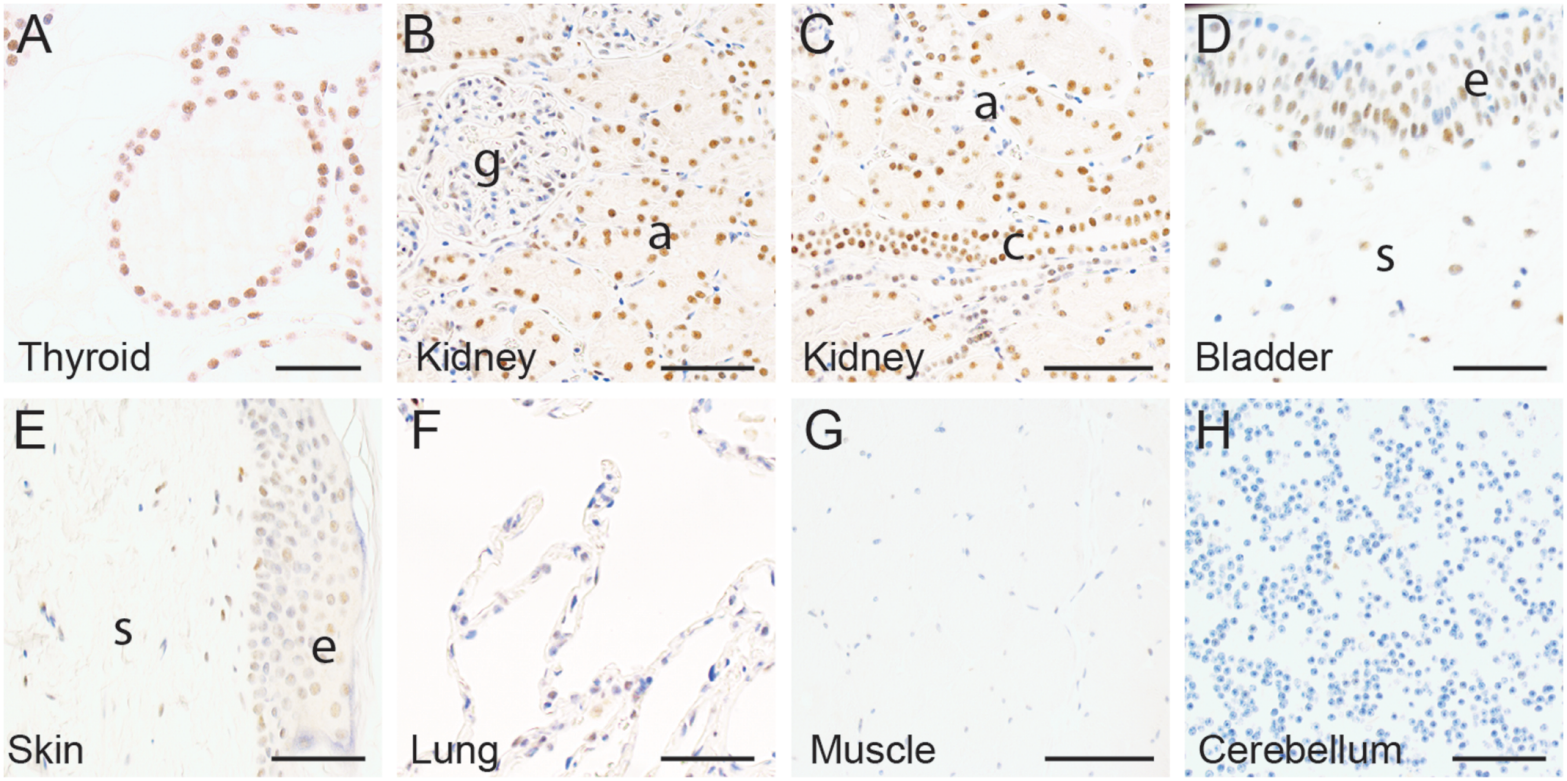
Expression of ZBED1 in other tissues. (A) thyroid, (B-C) kidney, (D) bladder, (E) skin, (F) lung, (G) muscle, and (H) cerebellum. g = glomerulus, a = acini, c = collecting ducts, s = stroma, e = epithelial. Scale bars = 50 μM.

We also investigated ZBED1 expression in a number of other tissues. Medium expression was detected in most cells of the follicular and parafollicular cells of the thyroid (Fig 6A). In the urinary tract, medium ZBED1 expression was seen in all epithelial cells of the proximal and distal convoluted tubules and in collecting tubules (Fig 6B). Low expression was evident in cells of the parietal layer and glomerulus of the renal corpuscle (Fig 6C). Medium ZBED1 expression was also present in the basal cells of the bladder epithelium and in a subset of stromal cells (Fig 6D). In the epidermis, most cells of the epithelia and some cells of the subjacent connective tissue were weakly positive (Fig 6E). In the lung, a smaller number of epithelial cells were weakly positive (Fig 6F). Muscle (Fig 6G) and cerebellum (Fig 6H) were completely negative for ZBED1 expression.

### ZBED1 expression is not associated with cell proliferation

**Fig 7:**
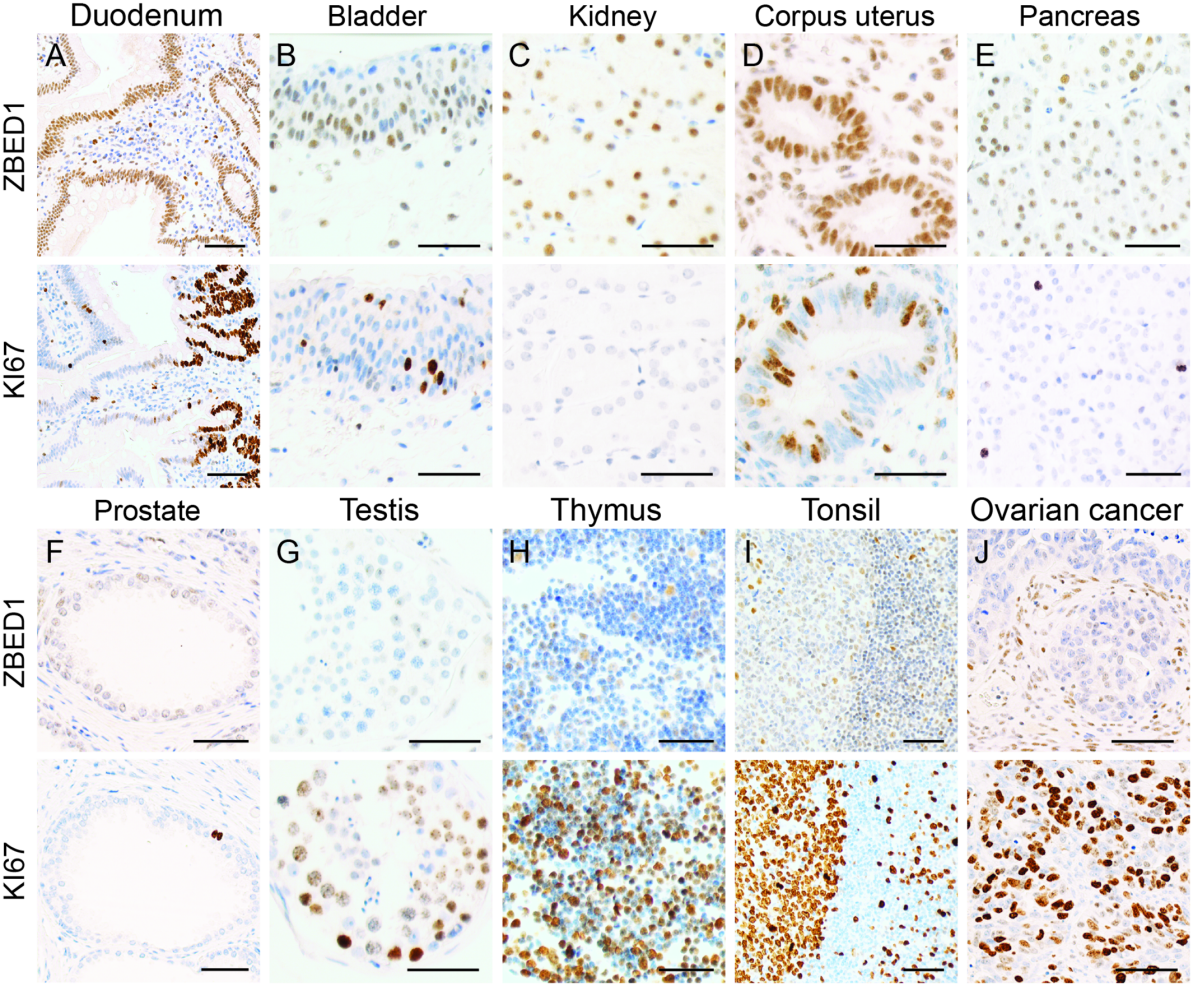
Comparison between ZBED1 and Ki-67 expression in different tissues. (A) duodenum, (B) bladder, (C) kidney, (D) corpus uterus, (E) pancreas, (F) prostate, (G) testis, (H) thymus, (I) tonsil, (J) ovarian cancer. Scale bars = 50 μM.

The *Drosophila* orthologue of ZBED1 has been associated with cell proliferation by upregulation of the expression of several genes important for DNA replication and cell cycle progression [2, 3, 11–15], as well as required for efficient progression through the late G1 and S phases of the cell cycle in in vivo studies [3]. To test for an association of ZBED1 expression with cell proliferation in human tissues, we compared ZBED1 and Ki-67 immunostainings of parallel tissue sections. Ki-67 was found to be present in the nuclei of cells during all active phases of the cell cycle, but was absent from resting cells, making this protein a widely used marker for cell proliferation [25]. We found that although ZBED1 and Ki-67 exhibited some overlap in cellular expression, ZBED1 was much more widely expressed in many tissues. For instance, in the epithelia of the duodenum and colon (Fig 7A), Ki-67 expression was limited to cells of the crypts, as expected, whereas ZBED1 expression was evident in most cells. A similar pattern was seen in the bladder (Fig 7B), kidney (Fig 7C), uterus (Fig 7D), pancreas (Fig 7E), prostate (Fig 7F) and glandular submandibularis (not shown), where Ki-67-positive cells constituted less than 20% of epithelial tissues and ZBED1 was expressed in most or all cells. On the other hand, cell types with high proliferative capacity and high Ki-67 expression, such as the spermatogonia of the testis tubules (Fig 7G) or lymphocytes of thymus (Fig 7H) and tonsils (Fig 7I), exhibited no or little ZBED1 expression. In agreement with this, the Ki-67 labelling index of cancers did not correlate with fractions of ZBED1-positive cells (Fig 7J). These results suggest that ZBED1 expression is not a hallmark of proliferating cells.

### ZBED1 expression in cancer

**Fig 8:**
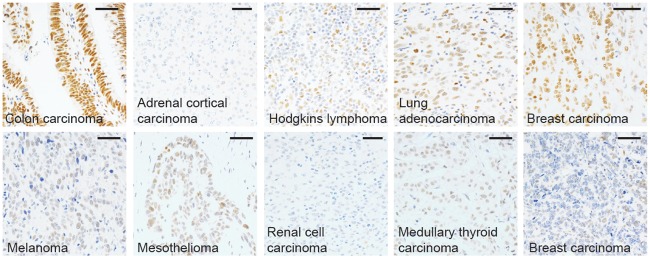
Expression of ZBED1 in tumors. The expression of ZBED1 was investigated in a mixed panel of solid tumors. Scale bars = 50 μM.

The *Drosophila* orthologue of ZBED1 has been suggested to play a role in tumorigenesis by regulating tumor suppressors and oncogenes [8]. Therefore, we examined the expression of ZBED1 in a panel of different tumor samples to investigate changes in ZBED1 expression associated with carcinogenesis (Fig 8). In some cases, the expression corresponded to that of the tissue of origin (e.g. colon, Hodgkins lymphoma and lung adenocarcinoma), but in other cases the expression was highly heterogenous (e.g. breast carcinomas, melanoma) or absent (e.g. renal cell carcinoma). These results suggest that ZBED1 expression may be subject to editing during malignant transformation, but a detailed analysis of expression levels in a larger cohort of tumors should be performed in a future study to validate this.

## Discussion

ZBED1 is the human orthologue of the *Drosophila* transcription factor dDREF. Although the function of dDREF is well described, little is known about the functions of ZBED1 in human cells and tissues. We have characterized the cellular expression pattern of ZBED1 in multiple human tissues to illuminate the cellular functions of ZBED1. We focused on a possible association between ZBED1 and cell proliferation since it is well-established that dDREF is important for cell proliferation.

Initially, we tested two different anti-ZBED1 monoclonal mouse antibodies on a panel of normal tissues and found that their staining profiles were highly similar, validating the specificity of the antibodies. Immunostaining of 24 normal tissues revealed that ZBED1 was widely expressed in the nuclei of cells derived from all three primary germ layers, in accordance with RNA-sequencing data from the Human Protein Atlas [26]. For instance, ZBED1 was found in all examined epithelial tissues. There was no apparent difference in expression level depending on the type of epithelia, but expression disappeared with differentiation in stratified squamous epithelium of the esophagus and transitional epithelium of the bladder. Similarly, ZBED1 expression was diminished with maturation of the columnar epithelia of the digestive tract. In cuboidal epithelium of glands and kidney all cells were positive.

ZBED1 was also widely expressed in stromal cells, including those of the gastrointestinal tract and female reproductive system. In most cases, only a subset of cells were positive, suggesting that ZBED1 expression may also be regulated with cell maturation in these cell types. In muscle cells, another cell type of the mesenchymal lineage, the expression of ZBED1 was diverse. In smooth muscle of the gastrointestinal tract, a subset of cells was ZBED1-positive, whereas skeletal muscle cells were completely negative.

Although, ZBED1 was found to be widely expressed, many types of parenchymal cells were negative, including liver, germ cells and cerebellum.

In various tissues (e.g. digestive tract, bladder and female reproductive tissues), it seems that the expression of ZBED1 disappears with cell maturation, suggesting that ZBED1 may potentially be involved in inhibition of cell differentiation. Additionally, strong expression of ZBED1 in female reproductive tissues (Fig 4A–4C), especially in the placenta, may suggest that ZBED1 is associated with fetal development in humans. This is similar to dDREF, which is involved in development-associated pathways in Drosophila [8].

Given the importance of dDREF in regulation of cell proliferation in *Drosophila*, we investigated whether ZBED1 was associated with cell proliferation in human tissues using Ki-67 as a proliferation marker. Although ZBED1 and Ki-67 clearly overlapped in some tissues, there were no indications of a correlation between the two. In most tissues, ZBED1 was much more widely expressed than Ki67 and was clearly expressed in multiple types of non-proliferating cells (e.g. bladder, kidney, corpus uterus, pancreas cells). In contrast, ZBED1 was much more restricted than Ki-67 in tissues with high cell proliferation (e.g. testis, thymus, tonsil). Thus, ZBED1 does not seem to be essential for cell proliferation in human tissues. These results suggest that ZBED1 has different functions than dDREF, and given that the two proteins share only 22% identity, differences in structure and function are indeed possible. In vitro experiments have shown that amino acids identified as important for DNA binding in dDREF are conserved in ZBED1 [18]. However, ZBED1 and dDREF bind similar, but not identical, DNA motifs, suggesting that the protein may exhibit different DNA binding in vivo. Furthermore, the in vivo chromatin binding of ZBED1 and dDREF likely depends on associated factors that may differ between these proteins due to structural differences important for protein interactions.

In conclusion, we explored ZBED1 expression in a large panel of human tissues illuminating its functions and providing the foundation for more definitive studies on the role of this protein in human cells and tissues. Our data clearly suggest that ZBED1 is not, as its *Drosophila* orthologue dDREF, a hallmark for cell proliferation. Findings of decreased ZBED1 expression with epithelial cell maturation and heterogenous expression in other cell populations instead indicate that ZBED1 may be involved in cell differentiation.
